# Opposing roles of *PIK3CA* gene alterations to EZH2 signaling in non-muscle invasive bladder cancer

**DOI:** 10.18632/oncotarget.14453

**Published:** 2017-01-02

**Authors:** Cristina Segovia, Mónica Martínez-Fernández, Marta Dueñas, Carolina Rubio, Fernando F. López-Calderón, Clotilde Costa, Cristina Saiz-Ladera, María Fernández-Grajera, José Duarte, Huberto García Muñoz, Federico de la Rosa, Felipe Villacampa, Daniel Castellano, Jesús M. Paramio

**Affiliations:** ^1^ Unidad de Oncología Molecular, CIEMAT (ed70A), Madrid, Spain; ^2^ Grupo de Oncología celular y Molecular, Hospital Universitario 12 de Octubre, Madrid, Spain; ^3^ Centro de Investigación Biomédica en Red de Cáncer (CIBER ONC), Spain; ^4^ Unidad Mixta Roche-CHUS, Hospital Universitario de Santiago de Compostela, Travesía de Choupana, s/n, Santiago de Compostela, A Coruña, Spain; ^5^ Departamento de Bioquímica y Biología Molecular I, Facultad de Biología, Universidad Complutense, Madrid, Spain; ^6^ Servicio de Anatomía Patológica, Hospital Universitario 12 de Octubre, Instituto de Investigación 12 de Octubre i+12, UCM, Av Cordoba s/n, Madrid, Spain

**Keywords:** bladder cancer, PIK3CA, genomics, Ezh2, recurrence progression

## Abstract

The high rates of tumor recurrence and progression represent a major clinical problem in non-muscle invasive bladder cancer. Previous data showed that EZH2-dependent signaling mediates these processes, whereas the frequent alterations of *PIK3CA* gene (copy gains and mutations) are predictive of reduced recurrence. Here we show, using clinical samples and bladder cancer cell lines, a functional interaction between EZH2- and PIK3CA-dependent signaling pathways. *PIK3CA* alterations mediated, on the one hand, the increased expression of two miRNAs, miR-101 and miR-138, which posttranscriptionally downregulate EZH2 expression. On the other hand, *PIK3CA* alterations facilitate the activation of Akt which phosphorylates EZH2 on Ser21, precluding the trimethylation of histone H3 in K27. Remarkably the increased expression of miR101 or miR138 and the expression of Ser21-phosphorylated EZH2 are good prognostic factors regarding non-muscle invasive bladder cancer recurrence and progression. Collectively, this study provides molecular evidences indicating that the gene expression rewiring occurring in primary bladder tumors, associated with increased EZH2 expression and activity and mediating the increased recurrence and progression risk, are prevented by PIK3CA-dependent signaling. This molecular process may have deep implications in the management of bladder cancer patients and in the design of novel molecularly targeted therapeutic approaches.

## INTRODUCTION

Bladder cancer (BC) is a complex disease caused by both genetic and environmental factors [1]. At diagnosis, two major types of BC: non-muscle invasive (NMIBC), and muscle invasive tumors (MIBC) are characterized. This pathological classification also defines the possible therapeutic options. The NMIBCs represent around 70% of BC and are treated by transurethral resections, in some cases followed by intravesical therapy. The MIBC are regularly treated by cystectomy followed by chemotherapy. Although NMIBCs have a favorable prognosis, they also show one of the highest rates of recurrence, which in some cases can progress into muscle-invasive tumors. This makes necessary a regular surveillance by cystoscopy and urine cytology indefinitely (EAU guidelines) [2]. As a consequence, NMIBC represents one of the most costly malignancies to health care systems in developed countries [2].

Genomic studies of BC identified different subtypes, and revealed that MIBC and NMIBC are characterized by distinct molecular profiles, suggestive of possible distinct molecular origin [3]. A large fraction of bladder cancers displays alterations in PI3K pathway components, including *PIK3CA* gene mutations frequently associated to low grade and stage tumors [4]. Remarkably, different mutations of the PIK3-mTOR pathway occur in NMIBC and MIBC, being the *PTEN* gene alterations more frequent in MIBC and associated with poor outcome [4]. These differences remain poorly understood, but they might define better therapeutic options, such as the increased sensitivity to mTOR inhibitors displayed by BC patients bearing mTOR-activating mutations [5]. Using a highly sensitive PCR technique, we have previously demonstrated that *PIK3CA* gene alterations are extremely frequent in NMIBC being present in non-affected bladder tissue, and associate with low recurrence and progression [6]. Conversely, genomic studies in human samples and in mouse models of NMIBC revealed that the recurrence and progression are associated with increased expression of *EZH2*, which promotes global changes in gene expression [7], including various miRNAs, involved in the repression of the epithelial mesenchymal transition [8] and the aberrant expression of lncRNA *HOTAIR* [8]. The different clinical evolution of tumors characterized by *PIK3CA* gene alterations and those exhibiting increased expression and activity of EZH2, led us to hypothesize that these two pathways may exert opposite roles in NMIBC. Here, we report that PI3K-dependent signaling negatively regulates EZH2 expression and activity in NMIBC, thus providing a possible explanation for the observed contrasting roles of these two pathways in this disease.

## RESULTS

### Transcriptional changes in NMIBC samples bearing *PIK3CA* alterations

The alterations in *PIK3CA* gene (mutations and copy gains or amplifications) are very frequent in NMIBC. We have previously shown in a series of 87 BC samples, using high sensitive PCR-based approaches, that the activating mutations (E545K/D, E542K and H1047R) or *PIK3CA* gene copy gains or amplifications may reach up to 50% of tumor samples, and they are also present in non-tumoral tissue from BC patients, suggesting field cancerization processes [6]. Moreover, they also seemed to associate with reduced recurrence likelihood [6]. We confirmed this association using updated clinical data from the same patients (Supplementary Figure 2A), and we also observed that these alterations are also associated with reduced progression upon recurrence (Supplementary Figure 2B). This suggests that such *PIK3CA* gene alterations are indicatives of good clinical outcome in NMIBC. To monitor whether similar association also occurs in MIBC, we used TCGA database. We observed that PIK3CA gene alterations were associated with increased overall and disease specific survival (Supplementary Figure 2C and 2D).

In order to gain insights into the possible biological basis of this reduced recurrence and progression, we analyzed our previous global transcriptome study [7] to discern possible gene expression changes that discriminate tumors bearing or not *PIK3CA* gene alterations. This provided 457 transcripts (306 overexpressed and 151 underexpressed) that characterized tumors bearing *PIK3CA* gene alterations (Supplementary Table 2).

**Figure 1:**
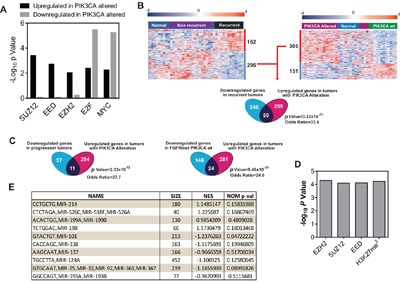
*PIK3CA* alterations oppose to EZH2 in NMIBC Transcriptome studies of human NMIBC recurrence. **A**. Summary of ChEA analyses showing enrichment for Polycomb binding motifs in genes identified between tumors with or without *PIK3CA* gene alterations. Note the enrichment in gene upregulated in tumors bearing *PIK3CA* alterations, whereas the binding by E2F and Myc are significant in upregulated and downregulated genes. **B**. Overlap between genes upregulated in tumors with *PIK3CA* alterations and downregulated in primary tumors showing recurrence. Left panel shows a Heatmap showing the distribution of genes (rows) and samples (columns) following supervised clustering (Pearson correlation and Average Linkage method) of 28 tumor and 10 normal samples according the recurrent and non-recurrent tumors. Right panel shows the corresponding heatmap showing the distribution according the presence or not of *PIK3CA* gene alterations in tumors. A red (overexpressed) to blue (downregulated) scheme following the above scale limits (in log_2_ scale) is shown. Lower panel shows a Venn diagram showing the overlap between genes downregulated in tumors that subsequently recurred and upregulated in tumors with *PIK3CA* gene alterations. Statistic significance was estimated by F Fisher’s exact test. **C**. Venn diagrams showing the overlap between genes downregulated in tumors that showed progression upon recurrence and upregulated in tumors with *PIK3CA* gene alterations (right panel); and overlap between downregulated genes in tumors bearing mutant *FGFR3* and wt *PIK3CA* and upregulated in tumors with *PIK3CA* gene alterations (right panel). Statistic significance was estimated by F Fisher’s exact test. **D**. Summary of ChEA analyses showing enrichment for Polycomb binding motifs in overlapping genes between those upregulated in tumors with *PIK3CA* gene alterations and downregulated in primary tumors showing recurrence. **E**. Summary of GSEA analyses showing enrichment for miRNA motifs in genes identified between tumors with or without *PIK3CA* gene alterations. NES: normalized enrichment factor. Negative values indicated enrichment in tumors bearing *PIK3CA* alterations.

Gene Ontology (GOBP; Supplementary Table S3 and Supplementary Table S4) and GSEA (Supplementary Table S5 and Supplementary Table S6) revealed that the upregulated genes in tumors bearing *PIK3CA* gene alterations played a major role in various metabolic and ribosomal processes, whereas the downregulated genes displayed an association with cytoskeleton organization and interphase cell cycle control. In addition, the ChEA analysis revealed a significant primary binding by various polycomb members in upregulated and downregulated genes (Figure 1A and Supplementary Table S7 and Supplementary Table S8). Since, gene downregulation mediated by polycomb repressing complexes appears to play a major role mediating recurrence in NMIBC [7], we decided to explore this observation. We found a highly significant overlap between the downregulated genes characteristic of recurrent tumors with those upregulated in tumors with *PIK3CA* gene alterations (Figure 1B). Moreover, the upregulated genes also displayed overlap with genes downregulated in tumors that showed progression upon recurrence (Figure 1C), and those downregulated in tumors bearing mutant *FGFR3* and wt *PIK3CA* (Figure 1C), which correspond to the group showing the earliest recurrence in our series [6]. Finally, we observed by ChEA analysis that the overlapping genes between downregulated in recurrent tumors and in those upregulated in tumors bearing *PIK3CA* alterations displayed significant enrichment in binding by polycomb members, and also in H3K27me3 marks (Figure 1D). Since the downregulated genes in recurrent tumors were predominantly associated with gene repression mediated by EZH2 [7], these observations might indicate that *PIK3CA* gene alterations would be acting opposite to EZH2-mediated gene repression. Moreover, GSEA analyses of *PIK3CA*-altered tumors identified various miRNAs, including those targeting EZH2 (Figure 1E), suggesting that *PIK3CA* alterations may affect the posttranscriptionally regulation of EZH2 expression through altered expression of miRNAs.

### mIR-101 and miR-138 levels define recurrence and progression in NMIBC

**Figure 2:**
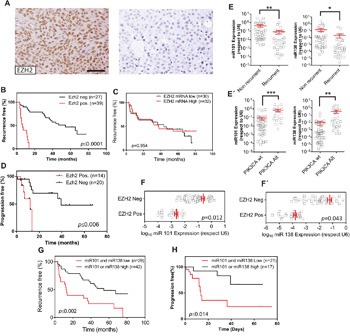
*PIK3CA* alterations oppose to EZH2 in NMIBC through miRNA expression Transcriptome studies of human NMIBC recurrence. **A**. Representative immunohistochemistry images of EZH2 staining showing positive (left panel) and negative (right panel). Bar=150μm **B**. Kaplan-Meier analysis showing that NMIBC patients with positive EZH2 expression (assessed by immunohistochemistry) in the primary tumor showed an earlier recurrence (*P* value was obtained by the log-rank test). **C**. Kaplan-Meier analysis showing that *EZH2* gene expression (mRNA values distributed according the median) in the primary tumor does not discriminate an earlier recurrence (*P* value was obtained by the log-rank test). **D**. Kaplan-Meier analysis showing that NMIBC patients with positive EZH2 expression (assessed by immunohistochemistry) in the primary tumor showed progression in recurrence (*P* value was obtained by the log-rank test). **E**. RTqPCR analyses showing the expression of miR-101 (left panel) and miR-138 (right panel) in recurrent and non-recurrent NMIBC samples Statistical significance was obtained by Mann–Whitney’s test. ** p value <0.01, * p value<0.05 E’) RTqPCR analyses showing the expression of miR-101 (left panel) and miR-138 (right panel) in NMIBC samples according the presence of alterations in PIK3CA gene in the primary tumors. Statistical significance was obtained by Mann–Whitney’s test. *** p value <0.005, ** p value <0.01. **F, F’**. RTqPCR analyses showing the expression of miR-101 (F) and miR-138 (F’) in NMIBC samples showing positive or negative EZH2 staining. Statistical significance was obtained by Mann–Whitney’s test. **G**. Kaplan-Meier analysis showing that NMIBC patients with low expression of miR-101 and miR-138 positive expression (according the median) in the primary tumor showed an earlier recurrence (*p* value was obtained by the log-rank test). **H**. Kaplan-Meier analysis showing that NMIBC patients with low expression of miR-101 and miR-138 positive expression (according the median) in the primary tumor showed progression in recurrence (*p* value was obtained by the log-rank test).

To explore the possible posttranscriptional regulation of EZH2, we monitored whether the expression of EZH2 protein (analyzed by immunohistochemistry) or mRNA (analyzed by RTqPCR) are equally predictive of clinical outcome. We found that tumors showing positive EZH2 staining dictated early recurrence (Figure 2A, 2B) and progression (Figure 2D), in agreement with our previous findings [7]. However, the increased *EZH2* gene expression levels (Figure 2C) was not predictive of early recurrence in NMIBC (Figure 2C), reinforcing a possible post-transcriptional modulation of EZH2. Of note, the positive EZH2 staining was not discriminative of tumor grade, stage, number of implants, tumor size or previous smoking history of the patients (Supplementary Figure 3).

We next studied possible differences in the expression of the miRNAs identified by the GSEA analysis between recurrent and non-recurrent tumors (Figure 1E). We found that only miR-101 and miR-138 displayed significant downregulation in recurrent respect to non-recurrent NMIBC samples (Figure 2E) and increased expression in tumors bearing *PIK3CA* gene alterations (Figure 2E’). Moreover, we also observed that the reduced EZH2 staining, indicative of reduced protein levels, associated with increased levels of miR-101 and miR-138 (Figure 2F, 2F’).

Since the increased protein levels of EZH2 predict early recurrence and tumor progression in recurrence (Figure 2B; see also [7]), we sought to determine whether miR-101 and/or miR-138 could also be predictive biomarkers of clinical outcome in NMIBC. We found that, although the individual expression of miR-101 or miR-138 were unable to predict the clinical outcome, tumors displaying increased expression of miR-101 or miR-138 showed reduced recurrence and reduced tumor progression upon recurrence better than those showing reduced expression of both miRNAs (Figure 2G, 2H).

### Expression of active Akt associates with Ser21 phosphorylated EZH2 in NMIBC

**Figure 3:**
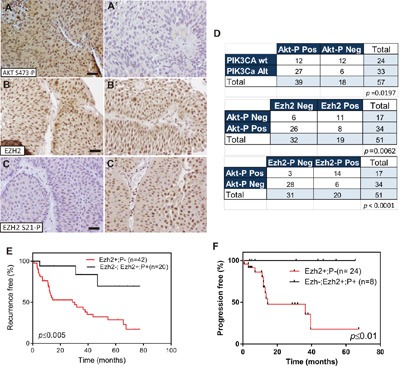
EZH2 phosphorylation in Ser21 is associated with *PIK3CA* gene alterations in NMIBC **A-A’**. Representative immunohistochemistry images of Ser473 phosphorylated Akt positive (A), and negative (A’), **B, C**. Representative immunohistochemistry images of primary tumors showing positive EZH2 (B) and negative Ser21 phosphorylated EZH2 (C) staining Bar=150μm B’, C’) Representative immunohistochemistry images of primary tumors showing positive EZH2 (B’) and positive Ser21 phosphorylated EZH2 (C’) staining Bar=150μm **D**. Contingency tables showing the significant association between phosphorylated Akt with tumors bearing PIK3CA alteration (upper panel), phosphorylated Akt (Ser473) with tumors showing negative EZH2 staining (mid panel) and phosphorylated Akt with tumors showing positive Ser21 phosphorylated EZH2 staining mutation (lower panel) **E**. Kaplan-Meier analysis showing that NMIBC patients with positive EZH2 and negative Ser21 phosphorylated EZH2 expression in the primary tumor showed an earlier recurrence (*p* value was obtained by the log-rank test). **F**. Kaplan-Meier analysis showing that NMIBC patients with positive EZH2 and negative Ser21 phosphorylated EZH2 expression in the primary tumor showed a tendency to display progression in recurrence (*p* value was obtained by the log-rank test).

The EZH2 catalytic activity to generate H3K27me3 chromatin marks is regulated by Akt-mediated phosphorylation [9]. Since *PIK3CA* gene alterations are associated with increased Akt activity in NMIBC [6], we monitored the expression of Ser 473 phosphorylated Akt (Figure 3A, 3A’), EZH2 (Figure 3B, 3B’) and Ser21-phosphorylated EZH2 (Figure 3C, 3C’) in NMIBC by immunohistochemistry. We observed a significant association of phosphorylated Akt with *PIK3CA* gene alterations and with reduced EZH2 protein expression (Figure 3D).

Importantly, we observed a predominant expression of Ser21 phosphorylated EZH2 in phosphorylated-Akt positive tumors (Figure 3D). Since the Ser-21 phosphorylation precludes EZH2 to carry out the H3K27me3 chromatin marks [9], and the downregulated genes that predict early recurrence preferentially displayed this chromatin modification [7], we tested whether Ser21-phosphorylated EZH2 could help to refine the recurrence probability in NMIBC patients. Of note, the positive Ser21-phosphorylated EZH2 staining was not discriminative of tumor grade, stage, number of implants, tumor size or previous smoking history of the patients (Supplementary Figure 4). Moreover, double immunofluorescence staining showed that all tumors positive for Ser-21 phosphorylated Ezh2 were also positive for total Ezh2 (Supplementary Figure 5). The series was divided into two groups: 1) those patients displaying positive EZH2 and negative Ser21-P EZH2 staining, and 2) those negative for EZH2, or those positive for both EZH2 and Ser21-P EZH2 (Supplementary Figure 5). Kaplan Meyer analysis of these two groups revealed that a statistically significant early tumor recurrence was associated with patients showing high EZH2 and negative Ser21-P EZH2 expression (Figure 3E). We also observed that progression in recurrences was only observed in primary tumors positive for EZH2 and negative in our series (Figure 3F). In order to confirm these observations, we analyzed, in a semiquantitive manner, the correlation between Akt activation (by AKT Ser473P staining) and the expression of total EZH2 or the Ser21-P EZH2 staining by the histoscore (HS) approach (see Materials and Methods) for each sample. This analysis revealed a significant negative correlation between AKT Ser473P and total EZH2, and a significant positive correlation between AKT Ser473P and Ser21-P EZH2 (Supplementary Figure 6). Moreover, we also observed that samples corresponding to tumors showing recurrence and those showing progression upon recurrence were predominantly associated with low HS for AKT Ser473P and Ser21-P EZH2 and high HS for total EZH2 (Supplementary Figure 6).

### PIK3CA opposes to EZH2 in BC cell lines

**Figure 4:**
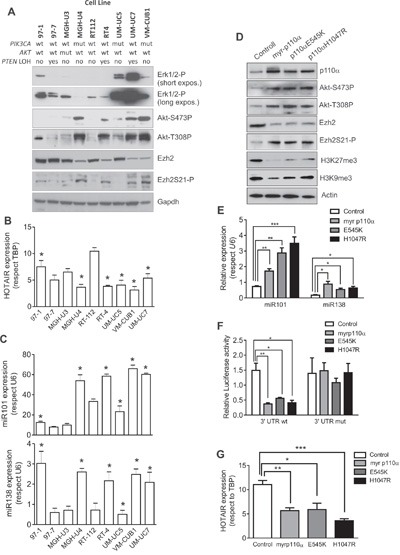
Expression of active PIK3CA mediates Ezh2 degradation and catalytic inhibition BC cells **A**. Immunoblot showing the expression of phosphorylated ERK1/2, phosphorylated Akt, total EZH2 and Ser 21 phosphorylated EZH2 in a collection of NMIBC cell lines, of known genomic charactersitics [10]. GAPDH was used to normalize loading. **B**. RTqPCR analyses showing the expression of the LncRNA *HOTAIR* in the BC cell lines. Asterisks denote those BC cell lines showing activated Akt according the immunoblot against Ser473 or Thr308 phosphorylated Akt (panel A). **C**. RTqPCR analyses showing the expression of miR-101 (upper panel) and miR-138 (lower panel) in the BC cell lines. Asterisks denote those BC cell lines showing activated Akt according the immunoblot against Ser473 or Thr308 phosphorylated Akt (panel A). **D**. Immunoblot showing the expression of p110α, Ser 473 and Thr308 phosphorylated Akt, EZH2, Ser21 phosphorylated EZH2, trimethylated K27 histone H3 and trimethylated K9 histone H3 in RT112 cells transfected with empty vector (control) or with plasmids coding for a myristoylated *PIK3CA* gene, or with E545K or H1047 *PIK3CA* gene. ACTIN was used to normalize the loading. **E**. qPCR analyses showing the expression of miR-101 and miR-138 (lower panel) in the RT112 cells transfected with the quoted plasmids. Statistical significance was obtained by Mann–Whitney’s test.*** p value <0.005, ** p value <0.01, * p value <0.05 **F**. Luciferase expression (normalized to Renilla values) of the construct containing the 3′UTR wt of EZH2 gene or a 3′UTR mutated in the seed sequences for miR-101 and miR-138 in RT112 cells transfected with the quoted plasmids. Statistical significance was obtained by Mann–Whitney’s test.** p value <0.01, * p value<0.05 **G**. qPCR analyses showing the expression of the LncRNA *HOTAIR* in the RT112 cells transfected with the quoted plasmids. Statistical significance was obtained by Mann–Whitney’s test.Test. *** p value <0.005, ** p value <0.01, * p value <0.05

To further substantiate the potential interaction between PI3K- and EZH2-dependent signaling, we selected a series of BC cell lines of low malignant potential and with complete molecular and genomic characterization [10]. By immunoblotting, we observed that the presence of *PIK3CA* mutations or *PTEN* gene LOH were associated with increased Akt Ser 473 and Thr 308 phosphorylation., We also observed that these cells also showed a trend to display partial reduction of EZH2 and increased Ser 21 phosphorylated EZH2 levels (Figure 4A). We found no relationship with activated ERK1/2. Interestingly, the activated Akt was partially and inversely correlated with the lncRNA *HOTAIR* expression (Figure 4B). This might be related with our previous observation suggesting that *HOTAIR* expression is, at least in part, under EZH2 positive regulation in BC [8]. On the other hand, the expression of miR-101 and miR-138 were, in general, increased in those cell lines showing activated Akt (Figure 4C). These results confirmed that the observed cross talk between PI3K- and EZH2-dependent signaling was also present in BC cell lines.

To demonstrate the converse effects between PI3K and EZH2, we expressed E545K, H1047R mutants or a myristoylated (constitutively active) *PIK3CA* gene constructs in RT112 cells. Their expression resulted in increased Akt activity, increased Ser-21 phosphorylation of EZH2, whilst reduced the total EZH2 and the H3K27me3 chromatin marks (Figure 4D). Moreover, we also observed that the different *PIK3CA* constructs also induced significant increase in miR-101 and miR-138 levels (Figure 4E) with non-significant differences among the different mutants. To monitor whether this increase in miRNA expression could account for the overall reduction in EZH2 levels, we performed a co-transfection experiment in which the different *PIK3CA* constructs were co-expressed along with pSiCheck2 plasmid in which the 3′UTR corresponding to EZH2 were placed downstream of the *Renilla* luciferase translational stop codon (Figure 4F). As a control, we used a similar construct including a 3′UTR of EZH2 in which the seed sequence for miR-101 and mir-138 has been mutated. We observed that the inclusion of the wt 3′UTR of EZH2, but not the mutant, led to a significant reduction of the luciferase activity. Similar results were obtained using the MGHU3 cells (data not shown). Finally, since we have observed that the expression of lncRNA *HOTAIR* is partially modulated by EZH2 in BC cell lines [8], we also monitored *HOTAIR* expression in the transfected cells. We observed that the *HOTAIR* levels were significantly reduced upon expression of E545K or H1047R mutants or a myristoylated *PIK3CA* gene constructs in RT112 BC cells (Figure 4G), although minor non-significant differences were detected among the different mutants, similar to those observed in the case of miR-101 or miR-138, were detected.

These results supported our observations of clinical samples, providing a possible molecular mechanistic framework to explain the opposite clinical outcome observed in NMIBC samples with respect to *PIK3CA* activation and EZH2-mediated gene expression reprogramming.

## DISCUSSION

The PI3K-Akt axis is a well-recognized oncogenic pathway in multiple tumor types, being considered a putative therapeutic target [11]. However, the significance of such alterations in relation to clinical outcome are not conclusive and may be related to the specific mechanism of activation (*PIK3CA* mutation or *PTEN* loss), or specific tumor subtypes. For instance, *PIK3CA* mutation associates good prognosis in breast cancer patients [12–14], and *PIK3CA* amplification predicts increased survival in colorectal cancer [15]. On the contrary, *PIK3CA* mutation favors malignant progression in head and neck squamous cell carcinoma [16], and poor prognosis in ovarian cancer [17], whereas PIK3CA amplification predicts reduced survival in gastric cancer [18]. To date, the relevance of PIK3CA alterations in bladder cancer recurrence remains poorly understood. Importantly, different types of alterations in PI3K-dependent signaling occur in NMIBC and in MIBC with potential different clinical consequences [4].

EZH2 aberrant expression is also frequent in multiple tumors thus becoming an attractive molecular target [19, 20]. The increased expression of EZH2 may be due to transcriptional and posttranscriptional mechanisms. In the first instance, *EZH2* transcription is positively modulated by E2F transcription factors [21], and in particular by E2F3 [7, 22]. In this regard, we reported the existence of a pRb-E2F-Ezh2 loop as an essential mechanism in bladder cancer *in vivo* in transgenic mice, which is also associated with poor clinical outcome in human BC patients [7]. Regarding the posttranscriptional regulation, EZH2 expression is controlled by various miRNAs [23]. In particular, increased miR-101 or miR-138 leading to reduced EZH2 expression have been reported for various types of cancer including BC [24]. However, the possible mechanisms of deregulated miR-101 and miR-138, have not been totally elucidated. In particular, reduced miR-101 expression has been associated with deletion [25, 26], and epigenetic silencing, probably mediated by c-myc or EZH2 increased expression [27]. To our knowledge, our data are the first evidence of positive modulation of these miRNAs by PI3K-dependent signaling.

In addition, EZH2 phosphorylation promotes its activation or repression depending on the specific residues phosphorylated [28]. Interestingly, the specific phosphorylation of EZH2 in Ser21 mediated by Akt suppresses its histone methyltransferase activity [9]. This could induce a change in the specificity of EZH2, which can methylate and activate other targets, such as androgen receptor [29], or Stat3 [30], and can mediate the response to chemotherapy [31]. This change in specificity has important clinical consequences, being associated with poor clinical outcome in prostate cancer [29] and glioblastoma [30], and explaining why in these tumors the increased Akt activity also associates with bad prognosis. Our data indicate that Ser21 phosphorylation of EZH2 is associated with low recurrence in NMIBC. This highlights the relevance of histone modification in the BC pathogenesis previously described through genomic studies [7, 32]. Nonetheless, our data cannot discard the existence of other possible mechanisms in advanced invasive BC.

Our previous data have suggested that Pi3K- and Ezh2-dependent pathways display opposite correlation with clinical outcome in BC [6, 7]. Here we provide a possible molecular explanation of these contrasting activities. which can be supported by our present data providing a possible molecular explanation. Activating mutations in *PIK3CA* gene, through increased expression of specific miRNAs, lead to reduced EZH2 expression. In addition, increased Akt activity, mediated in part by these *PIK3CA* gene mutations, produced the phosphorylation of EZH2 in Ser21, which accounted for reduced H3K27me3 marks.

Our present work may have potential clinical relevance. In particular, there is a current need of biomarkers that may predict early recurrence and progression of superficial NMIBC. Our data indicate that the presence of high levels of Ezh2 non phosphorylated in Ser21 (probably together with reduced expression of miR-101 and miR-138 and/or determination of *PIK3CA* gene status and activation of Akt) could represent excellent biomarkers, allowing a better identification of high risk tumors than the current clinical assessments [33]. However, this would require a large cohort studies and, if possible, quantitative measurements. In this regard, ongoing work will determine whether Ezh2 levels, together with the determination of specific miRNAs, can be used for prognostic determination in liquid biopsies [34]. In addition, our present data, together with the previous findings on the epigenetic changes occurring in BC, may also allow the identification of possible new therapeutic strategies [20].

Future work aimed to determine the possible activation of other transcription factors by EZH2-mediated methylation, and to ascertain whether similar processes occur in MIBC, could help to define better clinical intervention protocols for BC management.

## MATERIALS AND METHODS

### Patients

Tumor samples and medical records were analyzed from 87 patients who had been consecutively evaluated and treated by transurethral resection at the Urology Department of the University Hospital “12 de Octubre” between January 2009 and October 2011. Informed consent was obtained from all patients and the study was approved by the Ethical Committee for Clinical Research of University Hospital “12 de Octubre” (2014/0362). The sample recollection and preservation procedures have been reported elsewhere [6, 7]. The pathologic and clinical data of patients are provided in Supplementary Table 1. The determination of *PIK3CA* gene mutations and copy number in primary tumor sample DNAs have been previously described [6]. Progression upon recurrence was considered when the tumor, at any recurrence event, displayed increased stage and/or grade as monitored by the same pathologist.

### Tissue microarray (TMA) and immunohistochemistry

The aforementioned cases fixed in formalin (10%) and embedded in paraffin were included in two separate TMAs (1.5-mm core diameter), with at least two representative duplicate cores for each case [35], and constructed with a manual tissue arrayer (Beecher Instruments, Sun Prairie, WI) using a standard method [36]. The TMAs were stained with HE and were reviewed to confirm the presence of representative tumor tissue (at least 70% of tumor cells). Immunohistochemistry was performed essentially as previously described [6]. Antibodies used were anti phospho-Ser473-Akt (Cell Signaling), anti EZH2 (Abnova) and anti-phospho-Ser21-EZH2 (AbCam). Initially, the classification of samples was performed according the percentage of positive cells in each samples (Neg: 0-30%, Mid: 30-60% and High: 60-100%). However, in most cases no significant differences were observed between mid and high samples (Supplementary Figure 1). Accordingly, the mid and high samples were grouped for final determinations. Signal was amplified using avidin-peroxidase (ABC elite kit Vector) and peroxidase was visualized using diaminobenzidine as a substrate (DAB kit Vector). Negative control slides were obtained by replacing primary antibodies with PBS (data not shown). Double-blind scoring of the results and selection of the thresholds, internal controls for reactivity of each antibody, and tissue controls for the series were done according previously published methods [6]. Immunohistochemitry scoring (Histoscore) was calculated by a semiquantitative approach following a previously reported method [37]. Briefly, staining intensity (0, 1+, 2+, or 3+) and the percentage of cells for each intensity were determined in a high power fixed field. The histoscore, ranging from 0 to 300, was then calculated according the following formula:

[1 × (% cells 1+) + 2 × (% cells 2+) + 3 × (% cells 3+)]

### RTqPCR

Total RNA was isolated using miRNeasy Mini Kit (Qiagen) according to the manufacturer’s instructions and DNA was eliminated (Rnase-Free Dnase Set Qiagen). Reverse transcription was performed using the Omniscript RT Kit (Qiagen), TBP gene was used as reference gene for normalization using 50ng of total RNA and specific primers (EZH2: 5′-CCTGTCGACATGTTTTGGTC-3′; TBP: 5′-GTGTTTAAAATCTACATA-3′). PCR was performed in a 7500 Fast Real Time PCR System using Go Taq PCR master mix (Promega) and 1 μl of cDNA as a template. Melting curves were performed to verify specificity and absence of primer dimers. Reaction efficiency was calculated for each primer combination. The sequences of the specific oligonucleotides used have been reported elsewhere [7, 8]. To measure quantitatively the expression of miRNAs, RNA was extracted using the same method as for the genes. Reverse transcription was carried out from 10 ng total RNA along with miR-specific primer using the TaqMan® MicroRNA Reverse Transcription Kit (Applied Biosystems). PCR assays were performed using TaqMan® Gene Expression Master Mix and 7500 Fast Real Time PCR System (Applied Biosystems) as reported [38]. For normalization, we used RNU6B.

### Microarray data

To determine gene expression in samples bearing *PIK3CA* alterations, we used a previously reported whole dataset (GSE38264) [7]. The discrimination between tumors with or without *PIK3CA* gene alterations (copy gains, mutations or both) was obtained by a supervised analysis of differential gene expression using Student’s T test (Adjusted Bonferroni *p*-val≤0.01 using 1000 random permutations) in the Multiexperiment Viewer 4.5 (MeV 4.5) software [39]. Hierarchical clustering analysis was done using Pearson correlation and complete linkage method. To search for possible overlapping between the obtained upregulated gene signature and those genes downregulated in recurrent NMIBC samples previously reported [7], we used a Fisher's exact test, and was considered statistically significant for Odds Ratio ≥ 2 and *p* ≤ 0.05. Gene Ontology of Biological processes was performed using the DAVID webtool (https://david.ncifcrf.gov/) [40]. Gene Set Enrichment Analysis (GSEA) was performed using the MSignature and Motif databases [41]. Identification of transcription factor binding was performed using the ChiP enrichement analysis of the Enrich webtool (http://amp.pharm.mssm.edu/Enrichr/) [42, 43]

### Cell lines and plasmids

Cells were kindly provided by Dr. FX Real (CNIO, Madrid, Spain) and routinely cultured in DMEM supplemented with 10% FBS and 1% of antibiotic-antimycotic. Transfections were performed as previously described [8, 38]. Only Mycoplasma-free cultures were used. Plasmid coding for myr-p110α, E545K and H1047R PIK3CA mutants (in pBABE backbone) were generously provided by Dr. A. Carnero (IBIS/HUVR/CSIC/Universidad de Sevilla, Seville, Spain). Luciferase assays were performed using pSiCheck2 (Promega) plasmid derivatives after cloning the wt 3′UTR sequence of EZH2 gene (CTCGAGCTGCCTTAGCTTCAGGAACCTCGA GTACTGTGGGCAATTTAGAAAAAGAACATGCAGT TTGAAATTCTGAATTTGCAAAGTACTGTAAGAATA ATTTATAGTAATGAGTTTAAAAATCAACTTTTTATT GCCTTCTCACCAGCTGCAAAGTGTTTTGTACCAGT GAAGCGGCCGC), or its mutated counterpart (CTCGAGCTGCCTTAGCTTCAGGAACCTCGcgcgcggcGGGC AATTTAGAAAAAGAACATGCAGTTTGAAATTCTG AATTTGCAAcgcgcggcAAGAATAATTTATAGTAATGA GTTTAAAAATCAACTTTTTATTGCCTTCTgcgatcgTG CAAAGTGTTTTGTACCAGTGAAGCGGCCGC), into the XhoI-NotI sites of the expression vector downstream of the Renilla luciferase translational stop codon. This plasmid includes a firefly luciferase for normalization. The luciferase (Renilla and Firefly) activity was measured following the manufacturer’s recommendations.

### Western blot

Cell pellets were disrupted by freeze-thawing cycles in lysis buffer (200mM4-(2-hydroxyethyl)-1-piperazineethanesulfonic acid pH 7.9, 25% glycerol, 400mM NaCl, 1mM ethylenediaminetetraacetic acid, 1mM ethylene glycol tetraacetic acid, 1mg/ml aprotinin, 1mg/ml leupeptin, 1mM phenylmethanesulfonyl fluoride, 20mM NaF, 1mM NaPPi, 1mM Na3VO4, 2.5mM dithiothreitol, and centrifuged to obtain supernatant containing total protein. Thirty five micrograms of protein per sample were resolved by SDS PAGE (Invitrogen) and transferred to nitrocellulose membranes (Amersham). Membranes were blocked with 5% non-fat milk diluted in Tris-buffered saline (TBS) and incubated with the appropriate antibodies diluted in TBS-Tween 0.5% bovine serum albumin. Secondary antibodies were purchased from Jackson ImmunoResearch. Super Signal West Pico Chemiluminscence Substrate (Pierce) was used according to the manufacturer’s recommendations to visualize the bands. Antibodies used are mouse monoclonal anti EZH2 (Abnova), anti phosphoSer21-EZH2 (AbCam), anti phosphoSer473-Akt (Epitomics and Cell Signaling), anti phosphoThr308-Akt (Cell Signaling), anti K27 trimethylated (Millipore) or K9 trimethylated histone H3 (AbCam) and anti Phospho-ERK1/2 (Thr202/Tyr204-P; Cell Signaling). Loading was controlled by using an anti Actin or anti GAPDH antibody (Santa Cruz Biotechnology).

### Statistical analysis

Comparisons were performed using the Wilcoxon–Mann–Whitney test (for unpaired samples without normal distribution) and Student’s t Test (for paired samples showing normal distribution). Survival analyses (recurrence free or tumor progression in recurrence) according to various variables were performed using the Kaplan–Meyer method and differences between the patient groups were tested by the log-rank test. Contingency analyses were performed using the F Fisher’s exact test. Discrimination between samples showing increased or decreased tumor/normal relative expression of either gene or miRNA expression was made using the median. SPSS 17.0 and Graph prism 6.0 software were used.

## SUPPLEMENTARY FIGURES AND TABLES
















