# Supplementary material for: Opposing roles of PIK3CA gene alterations to EZH2 signaling in non-muscle invasive bladder cancer
Source: Oncotarget. 2017 Jan 2;8(6):10531–42. doi: 10.18632/oncotarget.14453 (PMC5354678; doi:10.18632/oncotarget.14453)
Supplement: Supplementary file 1 [file oncotarget-08-10531-s001.pdf]

# Opposing roles of *PIK3CA* gene alterations to EZH2 signaling in non-muscle invasive bladder cancer

## SUPPLEMENTARY FIGURES AND TABLES

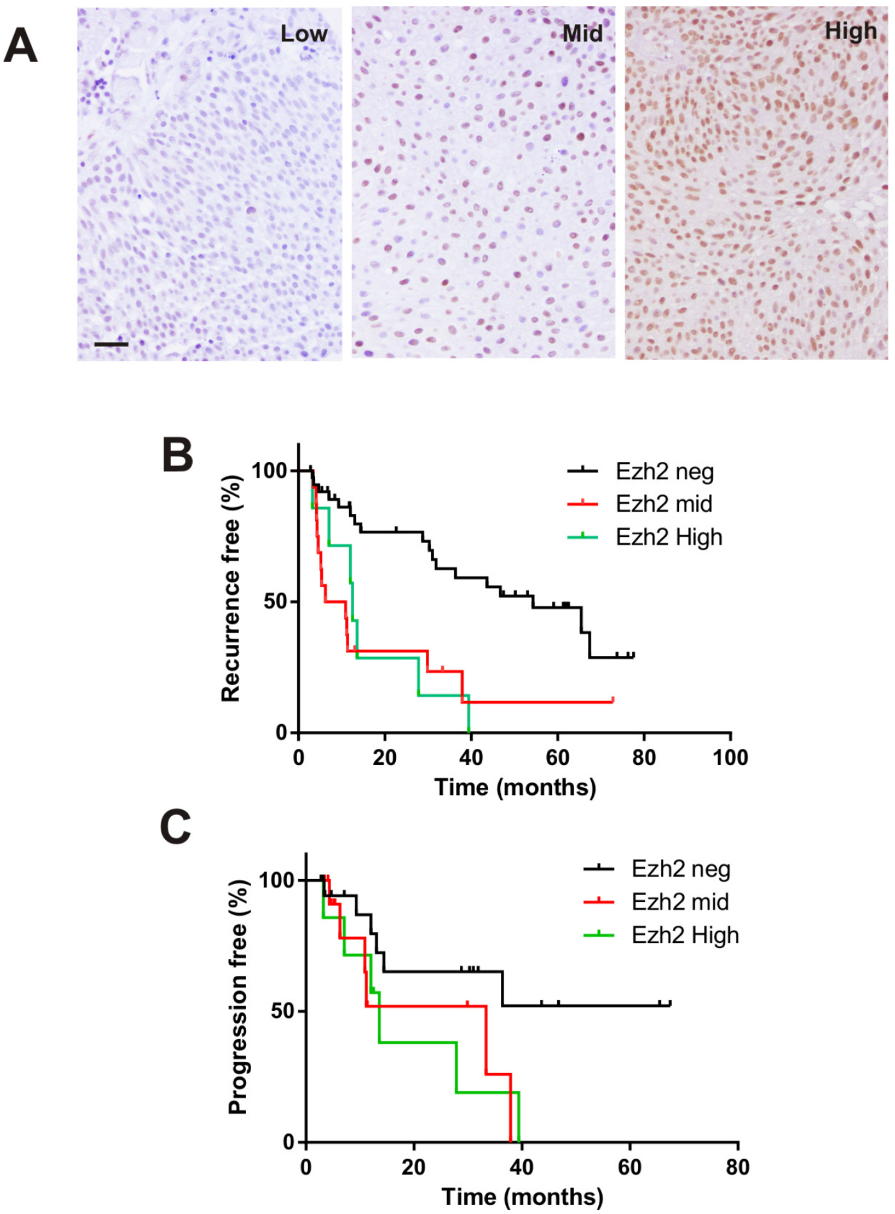


**Supplementary Figure 1: A.** Representative immunohistochemistry images of EZH2 staining showing the three main categories of staining: Low (0-30% of positive cells), Mid (30-60% of positive cells) or High (60-100% of positive cells). Bar=150µm. **B.** Kaplan-Meier analysis showing the NMIBC recurrence according the three categories of Ezh2 staining. The p Value estimated by Log rank for mid and High groups is not significant (p=0.457). **C.** Kaplan-Meier analysis showing the progression upon recurrence in NMIBC according the three categories of Ezh2 staining. The p Value estimated by Log rank for mid and High groups is not significant (p=0.295)

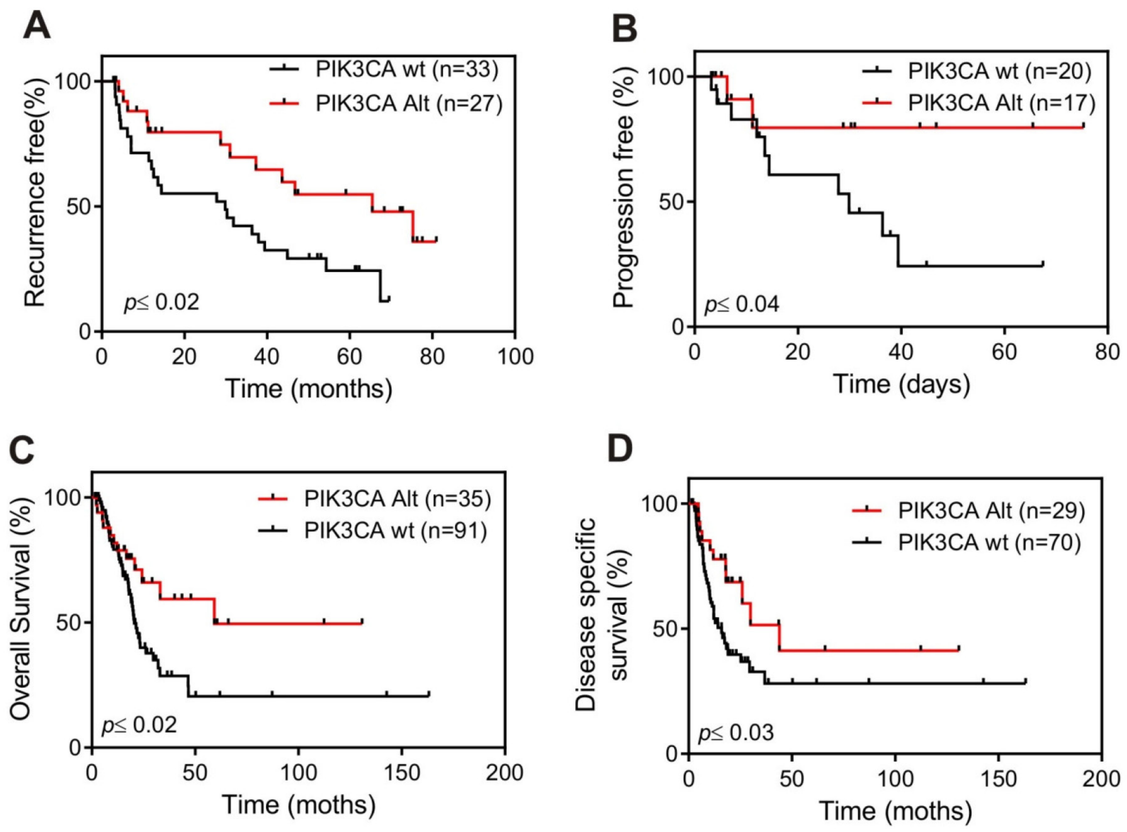


**Supplementary Figure 2: *PIK3CA* alterations represent a good prognostic factor in human BC.** **A-D.** Kaplan-Meier analysis showing that *PIK3CA* gene alterations (mutations and/or gene copy gains) associated with reduced recurrence **A.** and progression **B.** in NMIBC patients. PIK3Ca alterations also associated with increased overall survival **C.** and disease specific survival **D.** in BC patients from the cancer genome database (*P* value was obtained by the log-rank test).

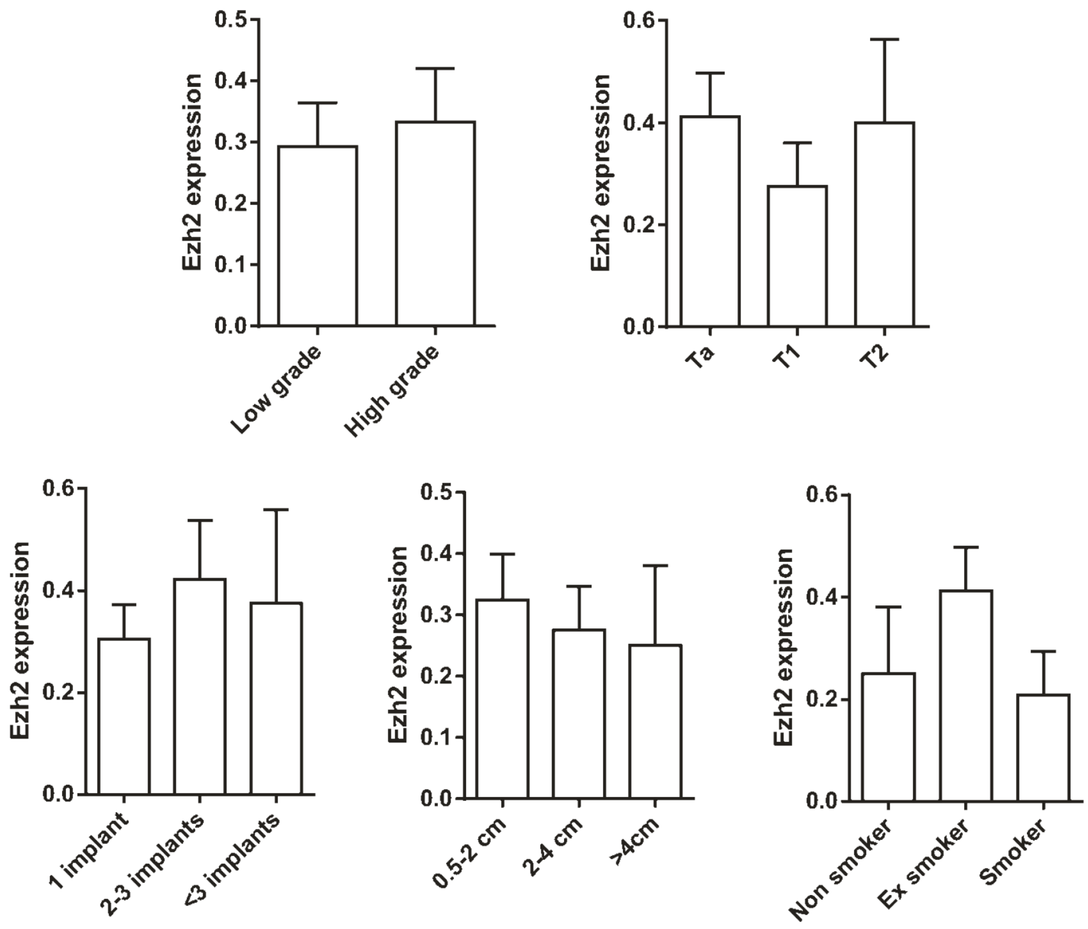


**Supplementary Figure 3: Distribution of EZH2 positive tumors according the tumor grade, stage, number of implants at diagnose, size of tumor or previous smoking history, showing no significant differences.**

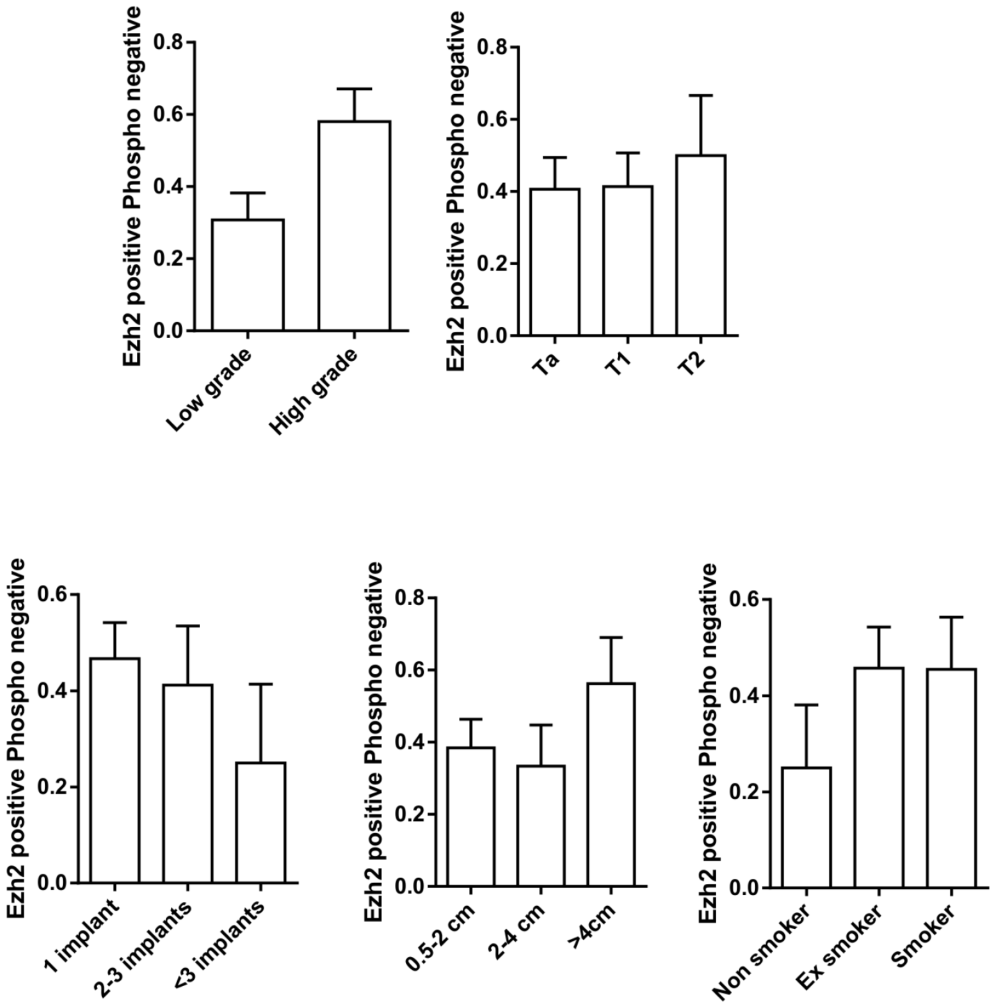


**Supplementary Figure 4: Distribution of positive Ser21-phosphorylated EZH2 staining according the tumor grade, stage, number of implants at diagnose, size of tumor or previous smoking history, showing no significant differences.**

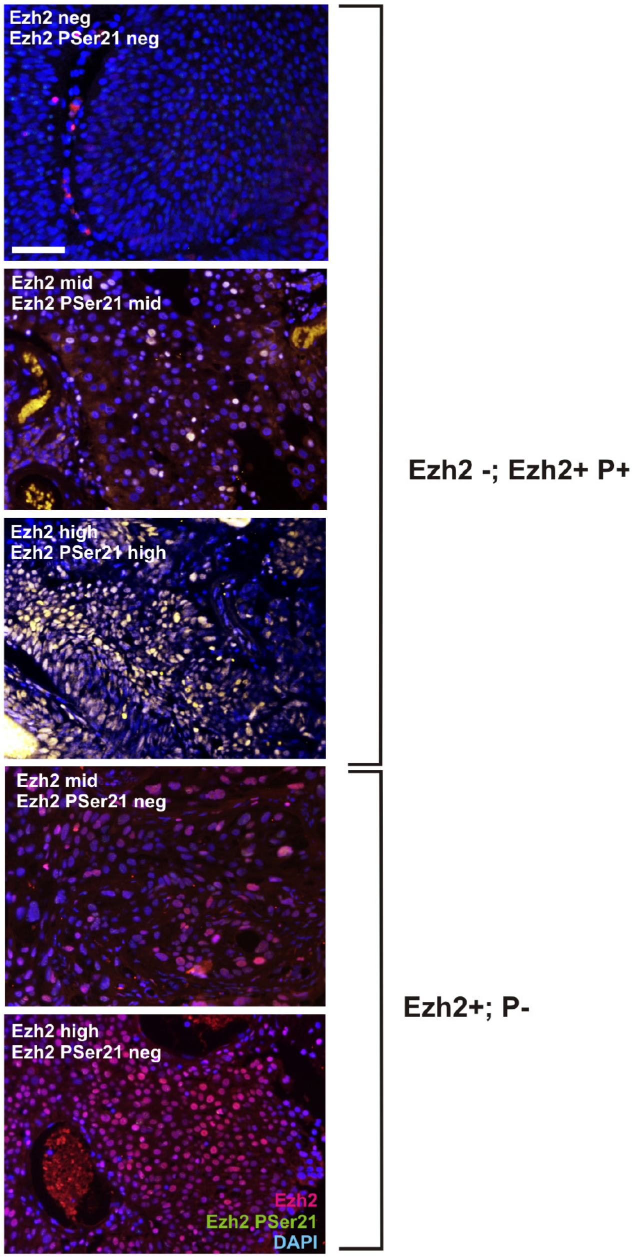


**Supplementary Figure 5: Representative images corresponding to double Immunofluorescence staining showing the expression of total EZH2 (red) and Ser21 phosphorylated EZH2 (green).** The examples also showed the different categories used for the classification of the tumors as represented in Figure 1E. DAPI (blue) was used to counterstain nuclei. Bar=150µm.

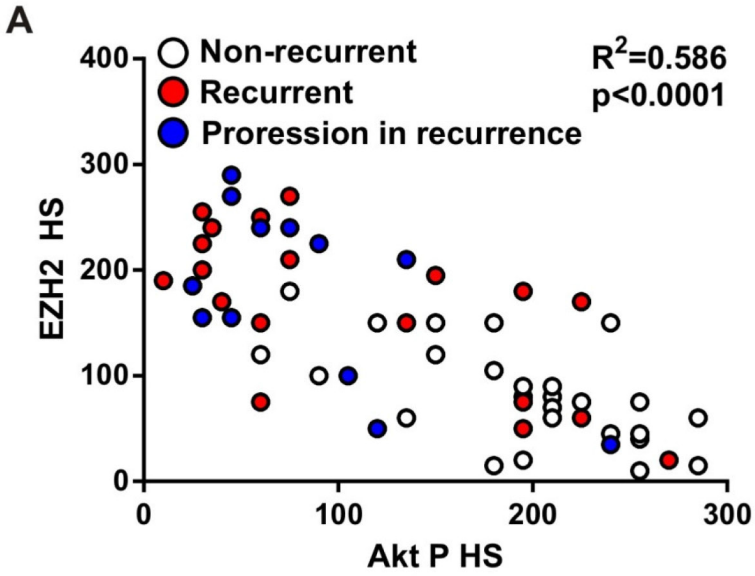


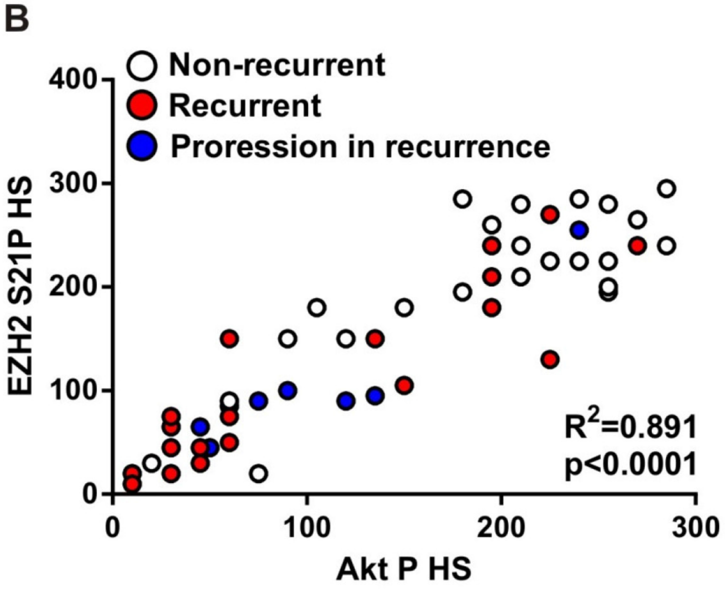


**Supplementary Figure 6: Correlation between phosphorylated Akt and total Ezh2 A. or Ser21 phosphorylated Ezh2 B.** The corresponding Histoscores were determined for each tumor sample as described in Materials and Methods. Primary tumor samples that afterwards showed recurrence or progression in the recurrence are identified. The correlation was determined by the Pearson method.

**Supplementary Table 1: Baseline Characteristics of the patients and Clinicopathological results in the series**

| **Patients (n)** | **87** |
|---|---|
| Age median (range) | 73.0 yr (49-90) |
| Sex | M=68<br>F=19 |
| Smoker status | No=15<br>Currently smoker=27<br>Ex smoker=43<br>ND=2 |
| Stage | Papilloma =1<br>Ta=40<br>T1=36<br>T2=10 |
| Grade | Low=45<br>High=37<br>PUNLMP=4<br>ND=1 |
| Alterations in normal mucosa | Dysplasia=6<br>Metaplasia =1<br>Glandular cystitis=1 |
| Follow up, median (range) | 48 months (3-81) |
| Tumor size, median (range) | 2cm (0.5-7) |
| Number of implants, median (range) | 1(1-10) |
| Specific death events | 10* |
| Non-specific deaths | 16** |
| Recurrence events | ND=10<br>No recurrence =35<br>Recurrence =42 |
| Time to recurrence, median (range) | 15.1 months (1.1-81) |
| Stage of recurrence | Ta=14<br>T1=16<br>T2 or higher=6<br>ND=6 |
| Grade of recurrence | Low=12<br>High=12<br>PUNLMP=1<br>CIS=2<br>ND=6 |
| Progression events*** | 16 |
| Treatment by local instillation | ND=23<br>Yes=15 (14 BCG; 1 mitomycin)<br>No=52 |

ND= Not determined
*All deaths due to progression of invasive bladder cancer.
**Unrelated to bladder cancer death causes umor in recurrence displays increased stage and grade
PUNLMP: Papillary urothelial neoplasia of low malignant potential
CIS: Carcinoma in situ

**Supplementary Table 2: Genes discriminating PIK3CA altered and PiK3CA wt NMIBC samples**

See Supplementary File 1

**Supplementary Table 3: Gene Ontology of Biological Processes of genes downregulated in PIK3CA altered compared to PiK3CA wt NMIBC samples**

See Supplementary File 2

**Supplementary Table 4: Gene Ontology of Biological Processes of genes upregulated in PIK3CA altered compared to PiK3CA wt NMIBC samples**

See Supplementary File 3

**Supplementary Table 5: Gene Set Enrichment Analysis (oncogenic pathways) of genes downregulated in PIK3CA altered compared to PiK3CA wt NMIBC samples**

See Supplementary File 4

**Supplementary Table 6: Gene Set Enrichment Analysis (oncogenic pathways) of genes upregulated in PIK3CA altered compared to PiK3CA wt NMIBC samples**

See Supplementary File 5

**Supplementary Table 7: ChIP (chromatin Immunoprecipitation) Enrichment Analysis (oncogenic pathways) of genes downregulated in PIK3CA altered compared to PiK3CA wt NMIBC samples**

See Supplementary File 6

**Supplementary Table 8: ChIP (chromatin Immunoprecipitation) Enrichment Analysis (oncogenic pathways) of genes downregulated in PIK3CA altered compared to PiK3CA wt NMIBC samples**

See Supplementary File 7
